# Functional analysis of the *GbDWARF14* gene associated with branching development in cotton

**DOI:** 10.7717/peerj.6901

**Published:** 2019-05-14

**Authors:** Ping Wang, Sai Zhang, Jing Qiao, Quan Sun, Qian Shi, Chaowei Cai, Jianchuan Mo, Zongyan Chu, Youlu Yuan, Xiongming Du, Yuchen Miao, Xiao Zhang, Yingfan Cai

**Affiliations:** 1 State Key Laboratory of Cotton Biology, Henan Key Laboratory of Plant Stress Biology, School of Life Sciences, Bioinformatics Center, School of Computer and Information Engineering, Henan University, Kaifeng, Henan, China; 2 College of Bioinformation, Chongqing University of Posts and Telecommunications, Chongqing, China; 3 Kaifeng Academy of Agriculture and Forestry, Kaifeng, China; 4 State Key Laboratory of Cotton Biology, Key Laboratory of Cotton Genetic Improvement, Cotton Institute of the Chinese Academy of Agricultural Sciences, Anyang, China

**Keywords:** GbD14, Cotton, Branch, Hormone, Strigolactone, Gene function

## Abstract

Plant architecture, including branching pattern, is an important agronomic trait of cotton crops. In recent years, strigolactones (SLs) have been considered important plant hormones that regulate branch development. In some species such as Arabidopsis, DWARF14 is an unconventional receptor that plays an important role in the SL signaling pathway. However, studies on SL receptors in cotton are still lacking. Here, we cloned and analysed the structure of the GbD14 gene in *Gossypium barbadense* and found that it contains the domains necessary for a SL receptor. The GbD14 gene was expressed primarily in the roots, leaves and vascular bundles, and the GbD14 protein was determined via GFP to localize to the cytoplasm and nucleus. Gene expression analysis revealed that the GbD14 gene not only responded to SL signals but also was differentially expressed between cotton plants whose types of branching differed. In particular, GbD14 was expressed mainly in the axillary buds of normal-branching cotton, while it was expressed the most in the leaves of nulliplex-branch cotton. In cotton, the GbD14 gene can be induced by SL and other plant hormones, such as indoleacetic acid, abscisic acid, and jasmonic acid. Compared with wild-type Arabidopsis, GbD14-overexpressing Arabidopsis responded more rapidly to SL signals. Moreover, we also found that GbD14 can rescue the multi-branched phenotype of Arabidopsis Atd14 mutants. Our results indicate that the function of GbD14 is similar to that of AtD14, and GbD14 may be a receptor for SL in cotton and involved in regulating branch development. This research provides a theoretical basis for a profound understanding of the molecular mechanism of branch development and ideal plant architecture for cotton breeding improvements.

## Introduction

Cotton is one of the most important economic crops. The architecture of cotton plants is determined primarily by their shoot branching patterns and flowering patterns, both of which directly affect cotton planting strategies, yield, planting area, mechanized harvesting suitability, and cotton planting costs (Reinhardt & Kuhlemeier, 2002; Sun et al., 2016). Research on plant architecture includes many aspects, among which branching development represents an important avenue of plant architecture research (Sun et al., 2018). Branch development is associated with the differentiation and development of the apical meristem, apical dominance, inflorescence development, stem growth, and lateral branch development (Wang, Smith & Li, 2018). Branch development is also affected by a variety of biotic and abiotic factors, including various hormones (Agharkar et al., 2007; Hayward et al., 2009; Lo et al., 2008), light (Leduc et al., 2014), and sugars (Granot, David-Schwartz & Kelly, 2013; Smeekens et al., 2010). In particular, with respect to plant hormones, auxin has a direct effect on plant branches (Domagalska & Leyser, 2011; Dun, Ferguson & Beveridge, 2006; Waldie, McCulloch & Leyser, 2014). In previous studies on Arabidopsis, rice and pea, gibberellin (GA) mutants were reported to have more branches than did wild-type (WT) plants (Lo et al., 2008; Silverstone et al., 1997).

Strigolactone (SL) is a type of plant hormone that was discovered recently. SL is involved mainly in the inhibition of both the development of axillary buds and the formation of plant branches (Gomez-Roldan et al., 2008; Yao, Li & Xie, 2018a). Interestingly, studies have shown that some mutant lines whose mutations affect the SL pathway, including the *ramosus* pea mutant, the *more axillary growth* Arabidopsis mutant, the *dwarf* rice mutant, and the *decrease of apical dominance* mutant of *Petunia*, exhibit no clear defects in growth but are highly branched. Breakthroughs have been made in the study regarding branch development (Arite et al., 2007, 2009; Beveridge, Dun & Rameau, 2009; Booker et al., 2004, 2005; Jiang et al., 2013; Liu et al., 2017; Snowden et al., 2005; Song et al., 2017; Sorefan et al., 2003; Stirnberg, Van De Sande & Leyser, 2002; Wang et al., 2015; Zhou et al., 2013; Zou et al., 2005, 2006). Studies on these genes have revealed that the mutations are caused by defects and SL deficiency or by mutations of the related genes involved in the SL signaling pathway.

The biological pathway of SL involves two main parts: the biosynthesis pathway and the signal transduction pathway. When the components of both pathways are mutated, the branch development process of the plants will be severely affected. For example, in rice, when the ubiquitination-associated degradation of D53 in the SL signaling pathway is blocked, the plant phenotypes exhibit increased branching (Jiang et al., 2013; Li et al., 2008; Liu et al., 2017; Wang et al., 2015; Zhou et al., 2013).

In the SL signaling pathway, there is a key factor, D14, that encodes a hydrolase. Studies have shown that this protein is a receptor for SL (Bennett & Leyser, 2014; Smith & Li, 2014; Yao et al., 2016). D14 is a non-classic receptor that functions as both an enzyme and a receptor, binding the active form of SL irreversibly (De Saint Germain et al., 2016; Fang & Chen, 2017; Yao et al., 2016). D14 has many homologues in different plant species, such as rice D14, Arabidopsis D14, *Petunia* DAD2, and pea RMS3, all of which belong to the α/β hydrolase family of proteins (Arite et al., 2009; De Saint Germain et al., 2016; Hamiaux et al., 2012; Nakamura et al., 2013; Waters et al., 2012; Yao et al., 2016). Previous studies have shown that, when the D14 gene is mutated, plant branch development is affected. For example, *d14-seto* mutant Arabidopsis plants exhibit a multi-branched phenotype (Chevalier et al., 2014).

In this study, to determine whether cotton branch development is also regulated by SL and to identify receptors for SL, the full length of the *GbD14* gene was cloned, and its structure was analysed. Moreover, we identified the function of *GbD14* and found that this gene plays an important role in the development of cotton branches, which is of great significance for understanding the molecular mechanism of branch development and is beneficial for selecting ideal types of cotton varieties.

## Materials and Methods

### Materials and growth conditions

Arabidopsis (Columbia-0) seeds were sterilized with 0.1% HgCl_2_ for 5 min. The seeds were then washed several times with sterile water to completely remove the HgCl_2_ solution (if the seeds were not washed completely, they would not germinate). The seeds were then spotted on 0.6 Murashige and Skoog (MS) solid media or 0.8 MS solid media, incubated at 4 °C for 4 days, and then subjected to constant temperature (16/8 h light/dark photoperiod, light intensity of 150 μmol m^−2^s^−1^, relative humidity of 80%). A total of 7 days after the seeds germinated, the seedlings were transferred to nutrient-enriched soil (vermiculite:nutrient soil = 1:1) and allowed to grow (The first 40 days involved a short light duration (12/12 h light/dark photoperiod), whereas the second 50 days involved a long light duration (16/8 h light/dark photoperiod); light intensity of 150 μmol m^−2^s^−1^; humidity of 80%).

Three cotton (*Gossypium barbadense* L.) varieties, Hai 1 (normal-branch cotton) and Xinhai 15 and 16 (nulliplex-branch cotton), were used. The cotton seeds were depilated and then soaked in water at 25 °C for approximately 8 h. The seeds were subsequently rinsed with water, after which they were wrapped in moist cotton wool. The seeds were then placed in a dish, which was incubated at 25 °C for 24 h. A total of 3 days after the seeds germinated, the seedlings were transferred to nutrient-enriched soil (perlite:nutrient soil = 1:2) and allowed to grow (16/8 h light/dark photoperiod, light intensity of 150 μmol m^−2^s^−1^, humidity of 75%).

### Preparation of cDNA and gDNA

The total RNA of 0.1 g of plant material was extracted with an EASY Spin Plus kit (Aidlab, Beijing, China) and reverse transcribed into cDNA using a Prime Script™ RT Reagent Kit (TaKaRa, Beijing, China). The cDNA was subsequently stored at −80 °C until use.

In order to prepare gDNA, young leaves were selected as materials, and their gDNA was extracted with a Plant Genomic DNA Kit (TianGen, Beijing, China), after which the gDNA was stored at −20 °C until use.

### Cloning of *GbD14*

The cDNA sequence of the *GbD14* gene was obtained from the cotton database (https://www.cottongen.org/blast) alignments, and specific primers (GbD14F: 5′-ATGGCAAACACCCTTTTAGAAG-3′; GbD14R: 5′-CTAGAAAACTCACCGCGGAAG-3′) were designed using the software Primer Premier 6.0. Using the cDNA of Xinhai 15 as a template, we amplified the target *GbD14* sequence via TransTaq DNA Polymerase High Fidelity (TransGen, Beijing, China).

### Phylogenetic trees and structure prediction

The amino acid sequence of GbD14 was derived via open reading frame (ORF) Finder (https://www.ncbi.nlm.nih.gov/orffinder/), and the primary structure of the protein was analysed with the online software Predict Prot. With the exception of the sequence of OsD14, which was retrieved from a rice database (ID: Os03g0203200), most of the sequences used for sequence homology alignment and phylogenetic tree construction were retrieved from the NCBI database. DNAMAN was used for multiple sequence alignment, and MEGA7 was used for phylogenetic tree construction (Accession numbers of D14 in difference species are as follows: ZmD14 (XP_008660429.1) from *Zea mays*, AtD14 (NP_195463.1) from *Arabidopsis thaliana*, BnD14 (XP_013731709.1) from *Brassica napus*, CpD14 (XP_021893497.1) from *Carica papaya*, CsD14 (XP_010517275.1) from *Camelina sativa*, DzDAD2 (XP_022752585.1) from *Durio zibethinus*, GbD14 (KP295470) from *Gossypium barbadense*, GhD14 (XP_016717728.1) from *Gossypium hirsutum*, GmD14 (NP_001347076.1) from *Glycine max*, GrD14 (XP_012451974.1) from *Gossypium raimondii*, HbDAD2 (XP_021676516.1) from *Hevea brasiliensis*, HvD14 (AJP07999.1) from *Hordeum vulgare subsp. vulgare*, NaDAD2 (XP_019258478.1) from *Nicotiana attenuate*, PhDAD2 (AFR68698.1) from *Petunia x hybrida*, PsDAD2 (XP_011017119.1) from *Populus euphratica*, PtDAD2 (XP_002302409.1) from *Populus trichocarpa*, RcDAD2 (XP_002510101.+1) from *Ricinus communis*, RsD14 (XP_018438965.1) from *Raphanus sativus*, TcD14 (XP_007018509.2) from *Theobroma cacao*). The predicted secondary structure of cotton GbD14 was derived from NPS (https://npsa-prabi.ibcp.fr/cgi-bin/npsa_automat.pl?page=/NPSA/npsa_seccons.html), and the 3D structure of GbD14 was obtained from RaptorX (http://raptorx.uchicago.edu/). Pymol software was used to superimpose the 3D structure of GbD14 and PhDAD2.

**Table 1:** Similarity analysis of nucleotide and amino acid sequences of D14 between *G. barbadense* and other species.

Species similarity	*Theobroma cacao* (%)	*Arabidopsis thaliana* (%)	*Petunia* (%)	*Oryza sativa* (%)	*Glycine max* (%)
Nucleotide	75.68	73.6	63.6	61.78	56.8
Amino acid	85.71	80.9	79.51	75.82	60.87

### Expression analysis

After the cotton plants produced buds, different tissues, including roots, stems, and leaves, were taken. Whole *Arabidopsis thaliana* seedlings were taken when they were mature enough for testing. The total RNA was extracted and then reverse transcribed into cDNA. The *GbUBQ7* and *AtACTIN8* genes were selected as internal reference genes, and Primer Premier 6.0 software was used to design specific quantitative primers (UBQ7F: 5′-GAAGGCATTCCACCTGACCAAC-3′; UBQ7R: 5′-CTTGACCTTCTTCTTCTTGTGCTTG-3′; ACTIN8F: 5′-ATCCTCCGTCTTGACCTTG-3′; ACTIN8R: 5′-TGTCCGTCAGGCAACTCAT-3′; GbD14F: 5′-TCCCAGGTTTCTCAATG-3′; GbD14R: 5′-CACGCAACACGGCACT-3′). The experiment was performed using an ABI 7500 Fast Real-Time PCR System with SYBR® Premix Ex Taq™ II (Tli RNaseH Plus) (TaKaRa, Dalian, China); the reaction procedure was 40 cycles of 95 °C for 20 s, 95 °C for 3 s, and then 60 °C for 30 s.

### Promoter analysis and β-glucuronidase histochemical assays

The upstream sequence of *GbD14*, which contains the promoter, was located in the *G. barbadense* database, and primers (*GbD14-GUS*F: 5′-GGGACAAGTTTGTACAAAAAAGCAGGCTACCACTGTTTCCGACACCACT-3′; *GbD14-GUS*R: 5′-GGGGACCACTTTGTACAAGAAAGCTGGGTCTTGGGTAGAGTTTGGAAGTTGA-3′) were designed by adding the *attB1* and *attB2* sequences to the 5′ ends of the original amplification primers. The promoter sequence (2,000 bp) was cloned by PCR amplification. The fragment was then cloned into a pKGWFS7.0 GUS fusion expression vector by BP and RP reactions, after which the recombinant vector was transformed into *Agrobacterium* (GV3101). WT *A. thaliana* plants were subsequently infected with *Agrobacterium*, and homozygous plants were obtained after three generations of kanamycin screening. We selected GUS seedlings or tissues at different growth stages and then examined them by chemical staining.

To prepare the GUS dye solution, one mg of X-gluc powder and one mL of dimethyl sulphoxide were added to an Eppendorf tube to prepare a solution whose final concentration was one mg mL^−1^. With respect to the dyeing process, the tissues or seedlings to be tested were first fixed in 90% acetone for 20 min, after which the material was washed several times with PBS. GUS dye solution was then added, after which the material was vacuumed for 15–20 min. The seedlings or tissues were then stained overnight at 37 °C. Afterward, the dye solution was discarded. The material was then washed several times with PBS, and at the end, it was washed with 70% alcohol until total discoloration was complete. The material was then examined under a microscope.

### Subcellular localization

Primers were designed by adding the *attB1* and *attB2* sequences to the 5′ ends of the original amplification primers. The ORF sequence of the *GbD14* gene was then amplified by primers (GbD14-GFPF: 5′-GGGGACAAGTTTGTACAAAAAAGCAGGCTACATGGCAAACACCCTTTTA-3′; GbD14-GFPR: 5′-GGGGACCACTTTGTACAAGAAAGCTGGGTCGAAAACTCACCGCG-3′). The fragment was cloned into a pK7FWG2.0 vector by BP and RP reactions; a *p35S*-GFP::GbD14 fusion expression vector was subsequently obtained. Both the recombinant vector and the control vector *p35S*-GFP were transformed into *Agrobacterium* (GV3101).

Leaves from normal tobacco plants were removed, after which the transformed *Agrobacterium* cells were injected into the tobacco leaves (See the previous method by Zhang Xiao) (Zhao et al., 2018). The leaves were then incubated in the dark for 2–3 days. Pieces were cut and removed from some of the affected leaves and were used to prepare slides. A drop of DAPI solution (Solarbio, Beijing, China) was added to the tissues, removed after 5 min, washed two to three times with PBS, and covered with a cover slip, then examined under a microscope.

### Construction of GbD14 transgenic plants

With respect to the construction of Arabidopsis overexpression plants, primers were first designed by adding *attB1* and *attB2* sequences to the 5′ ends of the original amplification primers. The ORF sequences of the *GbD14* gene were then amplified by primers (*GbD14*-OEF: 5′-GGGGACAAGTTTGTACAAAAAAGCAGGCTACATGGCAAACACCCTTTTA-3′; *GbD14-OE*R: 5′-GGGGACCACTTTGTACAAGAAAGCTGGGTCGAAAACTCACCGCG-3′). The obtained fragment was cloned into a pK7WG2.0 vector by BP and RP reactions, and a *GbD14* overexpression vector was then obtained. The recombinant vector was subsequently transformed into *Agrobacterium* (GV3101). WT *A. thaliana* plants were then infected with the transformed *Agrobacterium* cells, and homozygous plants were obtained after three generations of kanamycin screening.

To develop Arabidopsis complementation lines, primers were designed by adding *Kpn*I and *Bam*HI restriction enzyme cleavage sites to the original amplification primers. The ORF sequence of the *GbD14* gene was then amplified by primers (*GbD14*-REF: 5′-GGGTACCTGTTCATTCAACTTTC-3′; *GbD14-*RER: 5′-CGGATCCCTAGAAAACTCACCGCGGAAG-3′). After digestion, the target fragment was ligated into a Super 1300 vector by T4 ligase. The constructed 35S: GbD14 was subsequently transformed into *Agrobacterium* (GV3101), and the *A. thaliana Atd14* mutant (whose seed was generously donated by Zhao Liangjun, China Agricultural University) was infected with the transformed *Agrobacterium* cells. Homozygous transgenic plants were obtained after three generations of screening.

### Hormone treatments

When the cotton seedlings had developed three or four true leaves, the seedlings were sprayed with hormone solutions. The hormone solutions used included 100 μM auxin (indoleacetic acid), 50 μM abscisic acid (ABA) or GA, 100 mM jasmonic acid (JA), 50 mg L^−1^ brassinosteroids (BRs), and three μM GR24 (a synthetic SL). After different treatment times, the leaves of the cotton seedlings were cut with scissors and placed into a sterile bag, which was then quickly placed in liquid nitrogen for freezing. The material was subsequently stored at −80 °C or used immediately.

### Germination and root elongation experiments involving *A. thaliana*

Arabidopsis seeds were sterilized with 0.1% HgCl_2_ for 5 min. The seeds were subsequently washed several times with sterile water to completely remove the HgCl_2_ solution. The seeds were then spotted onto 0.6 MS solid media or 0.8 MS solid media, incubated at 4 °C for 3 days, and then subjected to constant temperature (16/8 h light/dark photoperiod, light intensity of 150 μmol m^−2^s^−1^, humidity of 80%). After the seeds germinated, the germination rate was determined daily, and a chart was constructed. However, after 4 days of germination, the seedlings were placed vertically on solid media that were supplemented with or without GR24 (12/12 h light/dark photoperiod, light intensity of 150 μmol m^−2^s^−1^, humidity of 80%). After 5 days of growth, the length of the main root was measured. After 10 days, the number of lateral roots were counted, and the length of the main root was measured again. The lateral root density, which is equal to the ratio of the number of lateral roots to the length of the primary root, was also recorded. A chart was ultimately constructed after statistical analysis.

## Results

### Characterization and phylogenetic analysis of the *GbD14* gene

By using the homologous cloning method, we obtained a key gene, *GbD14* (GenBank ID: KP295470), from *G. barbadense*. The gene is 810 bp in length and encodes 266 amino acids (Fig. S1A). This finding is essentially consistent with our predicted sequence information. We also analysed the nucleotide and amino acid sequences of the *GbD14* gene (Figs. S1A and S1B). By analysing the amino acid composition, we found that GbD14 is composed mainly of five kinds of amino acids: Leu (L), Val (V), Ala (A), Arg (R), and Gly (G). Several amino acids were relatively rare, including Trp (W), Met (M), Gln (Q), Lys (K), and Cys (C) (Fig. S1B).

**Figure 1:**
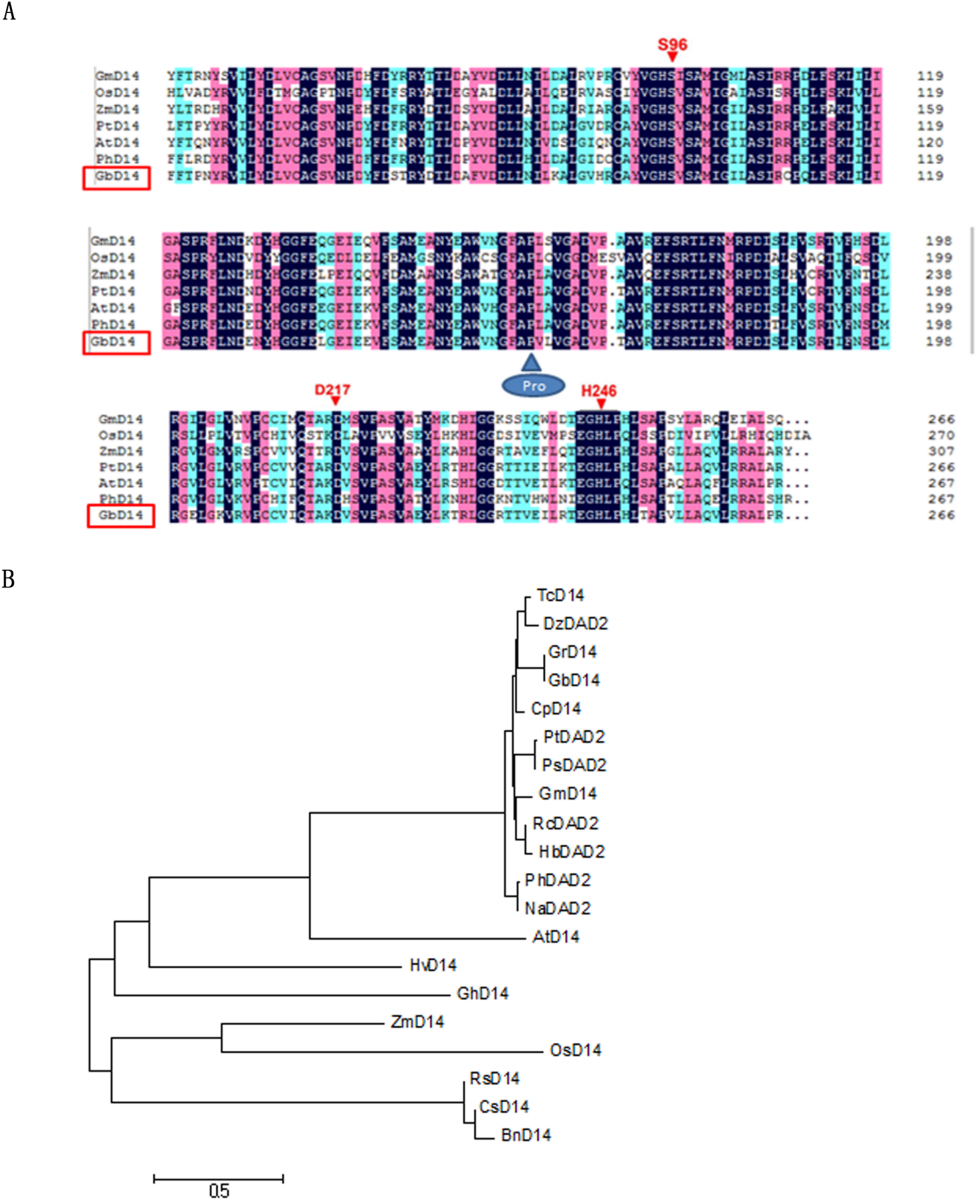
Characterization of GbD14. (A) Amino acid sequence alignment of GbD14 and other D14 (DAD2) proteins from other plant species, as performed by DNAMAN software. Identical amino acids are highlighted in blue. Three conserved amino acid sites (Ser96, Asp217, His246) are indicated by red arrows. (B) Phylogenetic relationship of the above D14 (DAD2) proteins. The evolutionary history was inferred using the neighbour-joining method. The analysis involved 20 amino acid sequences. All positions containing gaps and missing data were eliminated. There were a total of 212 positions in the final dataset. Evolutionary analyses were conducted via MEGA7.

We used the NCBI database to determine the nucleotide and amino acid sequences of the *D14* genes of several species (*Theobroma cacao*, *A. thaliana*, *Petunia*, *Oryza sativa*, *Glycine max*). First, the similarities between the nucleotide and amino acid sequences of the *D14* genes were compared. The comparison revealed that GbD14 in cotton was most homologous with that in cocoa; the amino acid similarity was 85.71%, and the nucleotide similarity was 75.68%. This homology was followed by that of Arabidopsis, whose amino acid sequence and nucleotide similarities were as high as 80.9% and 73.6%, respectively (Table 1). We then used the homologous sequence to perform an alignment with DNAMAN. The species we selected were *A, thaliana, O, sativa, P, trichocarpa, Z, mays, G, max*, and *Petunia x hybrida* (AtD14, OsD14, PtD14, ZmD14, GmD14, and PhD14). We found that these species have three conserved amino acid sites (Ser96, Asp217, His246) within an α/β hydrolase, which is consistent with previous reports. These three conserved amino acid sites are essential for the D14 protein in Arabidopsis and rice and are prerequisites for ensuring the function of the α/β hydrolase. As expected, these sites are also present in the sequence of GbD14 (Fig. 1A).

Similar to the amino acid and nucleotide sequence analysis, the protein sequences of D14 orthologues from 20 plant species were analysed, and the results showed that *GbD14* is highly conserved (Fig. 1B) and that the D14s of monocotyledons and dicotyledons are distinct. Compared with the D14s that belong to monocots such as *O. sativa* and *Z. mays*, GbD14 is more evolutionarily related to AtD14 and PhDAD2, which belong to dicots.

### Advanced structure prediction and analysis of GbD14

**Figure 2:**
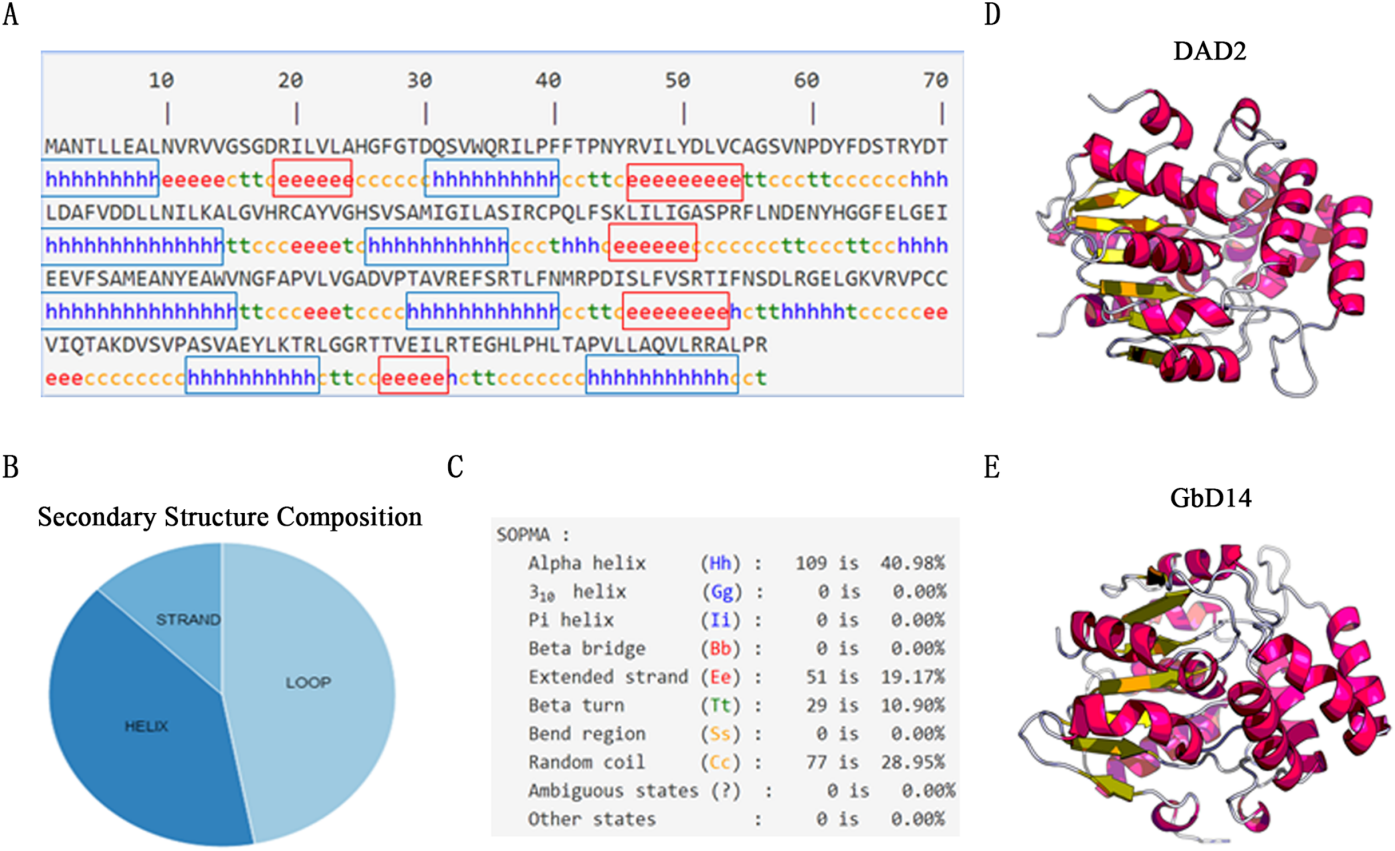
Advanced structure prediction of GbD14. (A) Distribution of *GbD14* secondary structure. “h” stands for alpha helix and is marked with a blue box. “e” stands for extended strand and is marked with a red square. (B) Composition of GbD14 secondary structure (strand, loop, helix). (C) Proportion of GbD14 secondary structure. The three-dimension structures of DAD2 (D) and GbD14 (E).

On the basis of the GbD14 amino acid sequence, the secondary structure was analysed using the online software SOPMA (Fig. 2A). The proportions of α-helices, β-folds, and random coils in the structure were 40%, 10%, and 29%, respectively (Figs. 2B and 2C). These three structures further formed a super-secondary structure, that is, the secondary structure of GbD14. Next, we used the online tool SWISS-MODEL to predict the 3D structure and found that the 3D structure of GbD14 is similar to that of the DAD2 protein in *Petunia*, which also belongs to the α/β hydrolase family, and had been reported as an SL receptor. By analysing the secondary and 3D structures of GbD14 (Figs. 2D and 2E), GbD14 and PhDAD2 were very similar (Fig. S2). We can conclude that the sequence and structure of GbD14 are similar to those of other D14 proteins from other species, which were reported as SL receptors.

### Promoter analysis of *GbD14* in transgenic Arabidopsis

**Figure 3:**
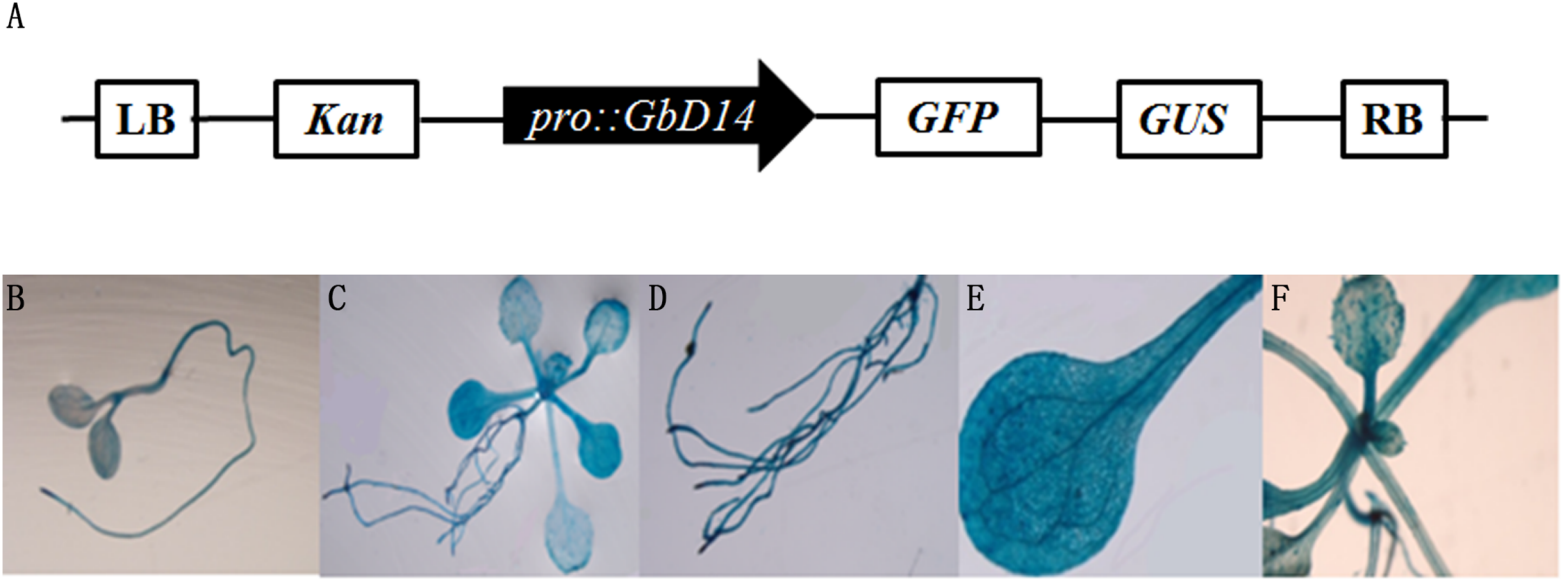
Promoter analysis of *GbD14* in Arabidopsis transgenic seedlings. (A) Schematic diagram of * ProGbD14::GUS*. Drawing is not to scale. Staining of 9-day whole transgenic seedlings (B), 20-day whole transgenic seedlings (C), staining of root (D), leaf (E), and rosette (F) tissues.

To analyse the tissue expression of the *GbD14* gene in Arabidopsis, we constructed a GUS fusion *ProGbD14::GUS* expression vector (Fig. 3A). This recombinant vector was transformed into *A. thaliana*. Afterward, we selected transgenic *A. thaliana* seedlings at different growth stages and examined them via chemical staining. The seedlings that grew for 9 days and 20 days were observed separately. A broad range of staining was observed. Most tissues exhibited coloration, especially the root tips, leaves, and vascular bundles were relatively dark. These findings indicated that *GbD14* is expressed mainly in these tissues (Figs. 3B–3F).

### Subcellular localization of GbD14

**Figure 4:**
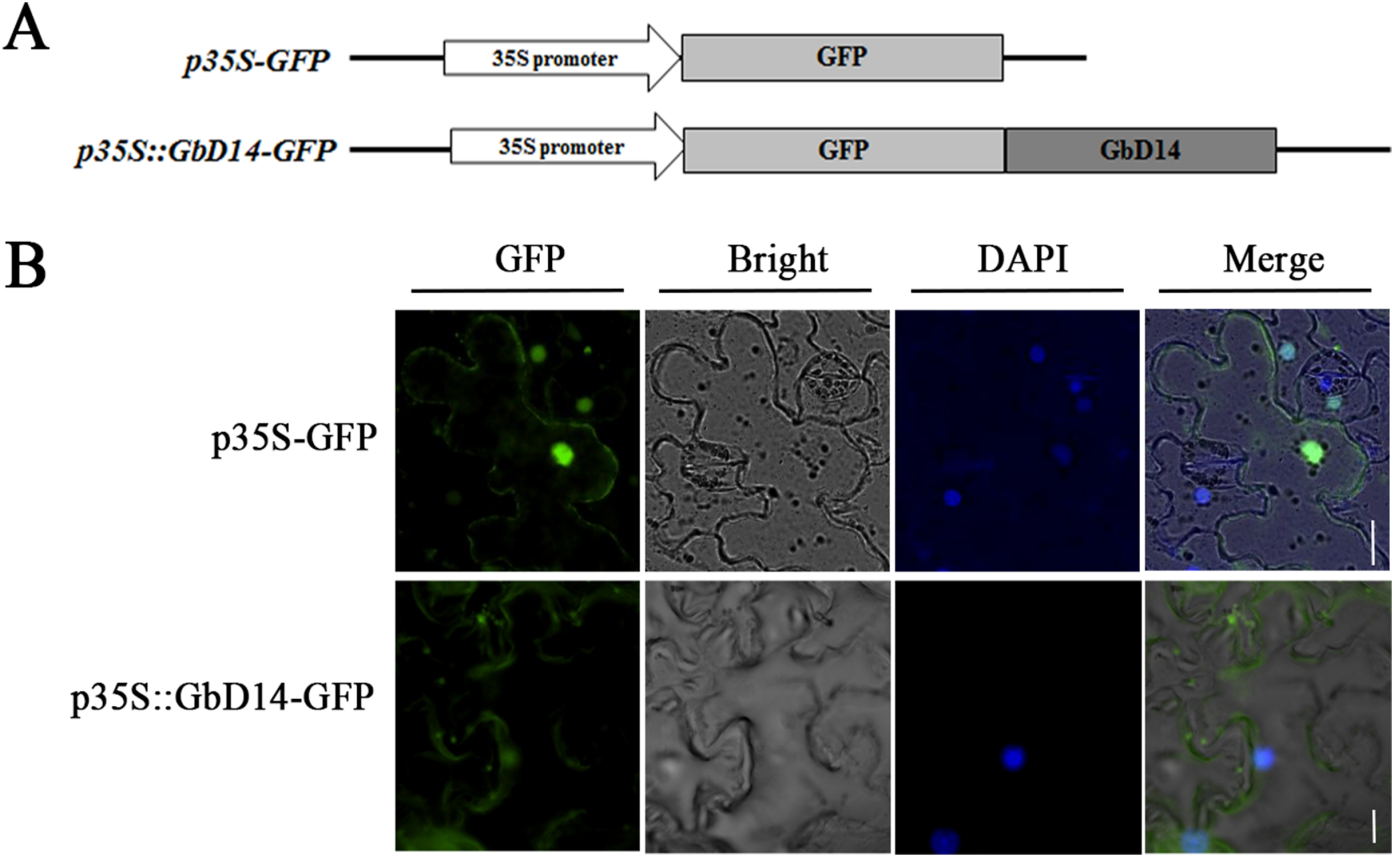
Subcellular localization of GbD14 in tobacco. (A) Schematic diagram of *p35S-GFP::GbD14*. Drawing is not to scale. (B) Subcellular localization of GbD14 in a tobacco epidermal cell. DAPI labels the location of nucleus. Transient GbD14-GFP fusion proteins was visualized 48 h after agroinfiltration. Bars, 20 μm.

To investigate the localization of GbD14 in cells, we constructed a *p35S-GFP::GbD14* fusion expression vector (Fig. 4A). The recombinant vector was transformed into tobacco, and the GbD14 protein with a GFP tag was expressed in tobacco cells. *p35S-GFP* was expressed throughout the tobacco cells, while *p35S-GFP::GbD14* was expressed in the cytoplasm and nucleus (Fig. 4B). These results suggest that GbD14 is localized mainly in the cytoplasm and nucleus.

### Expression patterns of *GbD14* in different fruit branch types of cotton

**Figure 5:**
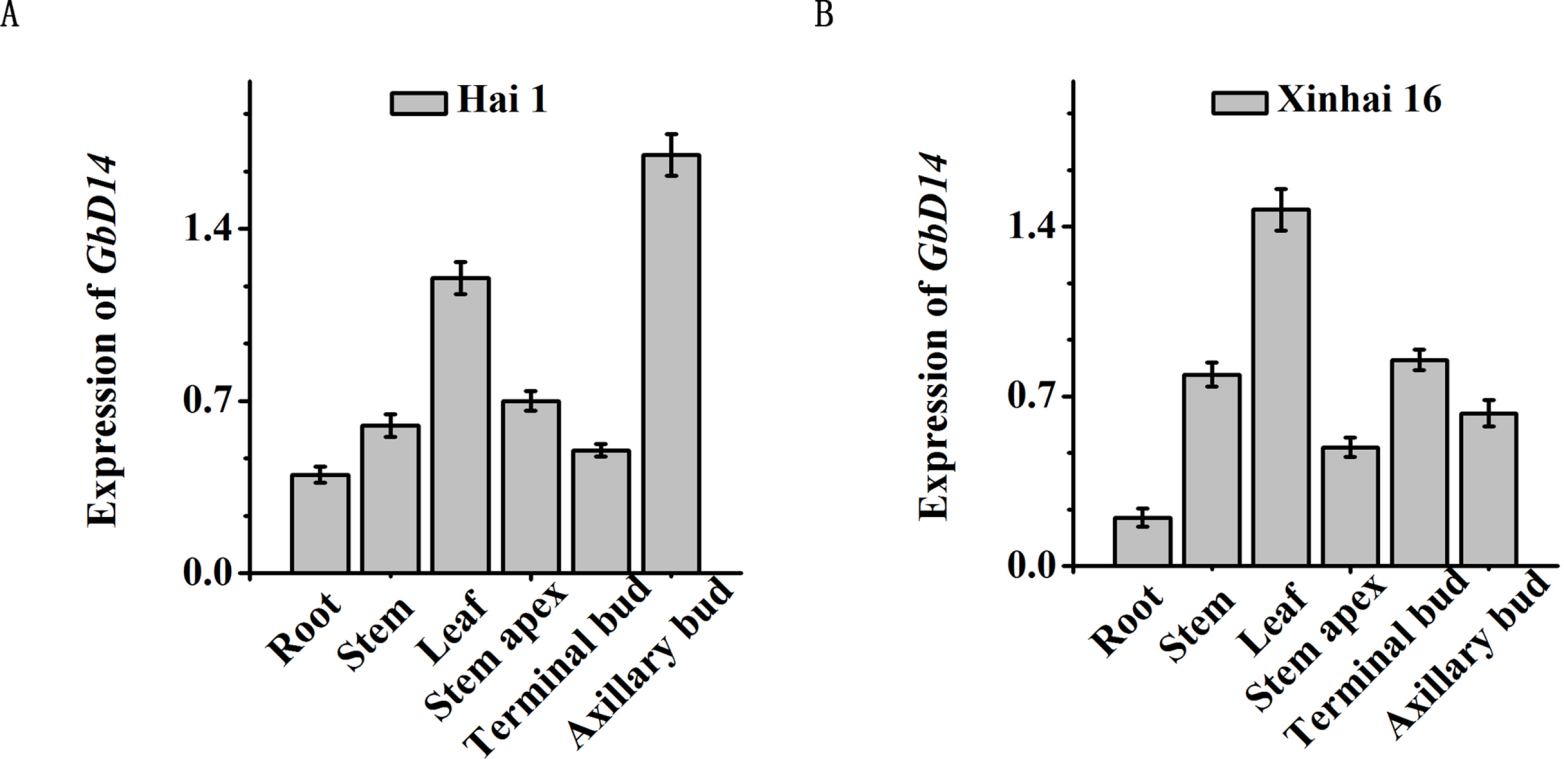
Analysis of the expression level of *GbD14* in different branching pattern varieties. The expression level of *GbD14* in Hai1 (A), in Xinhai16 (B). After growing the flower buds in the adult stage, the level in roots, stems, leaves, shoot tips, top buds, and axillary buds were tested. *GbUBQ7* was used as the internal control. Results are shown as mean ± SE of three replicates. A total of 30 plants were used per replicate. Experiments were performed three times with consistent results.

We analysed the expression patterns of GbD14 between two typical cotton branching types: nulliplex-branch and normal-branch cotton. There are natural differences in the formation of these two types of fruit branching patterns. To study whether GbD14 is related to the branching of fruit branches, we selected Hai 1 (normal-branch cotton) and Xinhai 16 (nulliplex-branch cotton) as materials. After the flower buds appearing, the roots, stems, leaves, shoot tips, top buds, and axillary buds were picked. We then examined the expression of *GbD14* in the two cotton varieties. *GbD14* was expressed in various parts, but there was a difference in expression between the two type varieties (Fig. 5). Between the two different fruit-branching varieties, *GbD14* was expressed primarily in the axillary buds in Hai 1 (Fig. 5A), while in the nulliplex-type, *GbD14* was expressed most in the leaves (Fig. 5B). Axillary buds are the key parts involved in controling the development of cotton branches. This difference in expression patterns suggests that GbD14 may be involved in the development of cotton fruit branches.

### GbD14 responds to SL and other plant hormones

**Figure 6:**
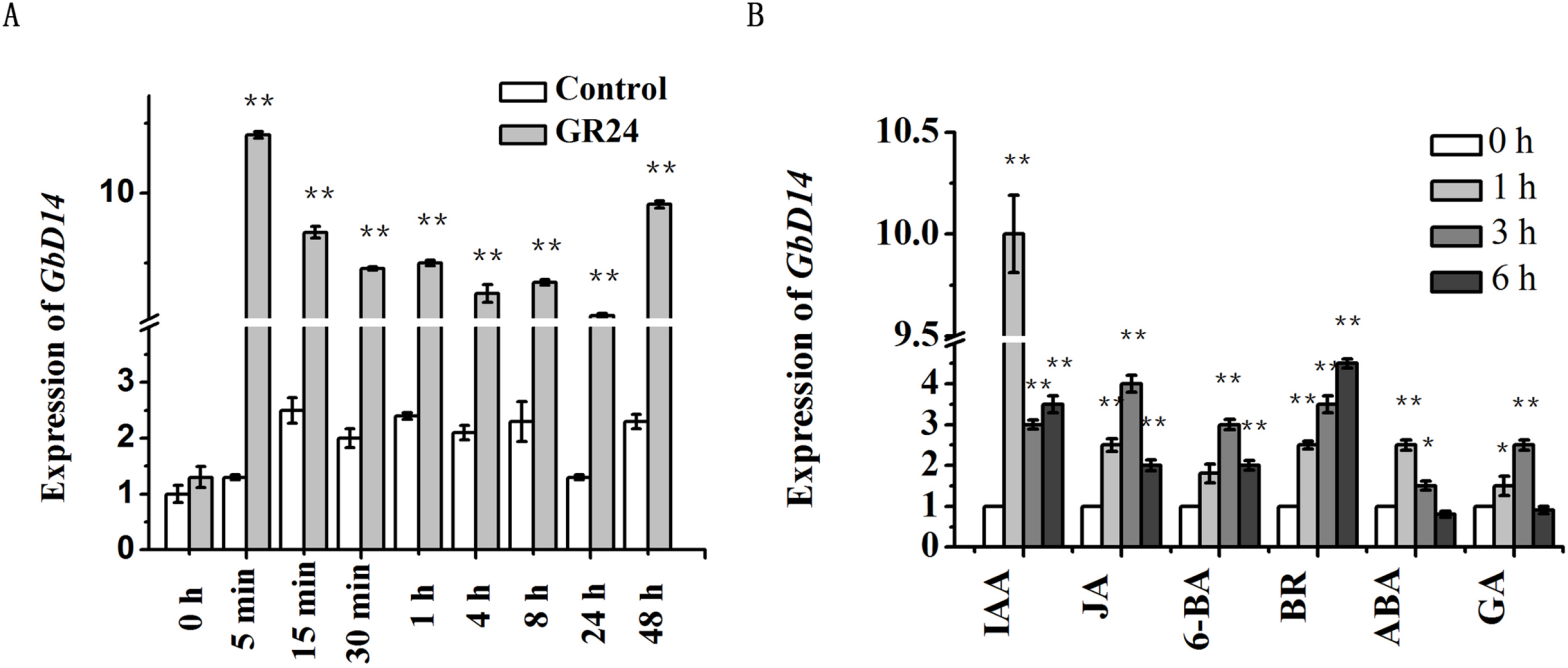
Analysis of the expression level of *GbD14* in Hai1 with plant hormones treatment. (A) The expression level of *GbD14* was tested with or without GR24 treatment 0 h, 5 min, 15 min, 30 min, 1 h, 4 h, 8 h, 24 h, 48 h. *GbUBQ7* was used as the internal control. (B) After hormones (IAA, JA, 6-BA, BR, ABA, GA) treatment 0, 1, 3, 6 h. Results are shown as mean ± SE of three replicates. Experiments were performed three times with consistent results. The single and double asterisks show significant differences between with GR24 (plant hormones) and without GR24 (plant hormones) by Student’s *t*-test at *P* ≤ 0.05 and *P* ≤ 0.01, respectively.

*D14* is a key gene involved in the SL pathway. To investigate whether *GbD14* can respond to SL, we investigated changes in *GbD14* expression levels. When the cotton plants reached the third-true leaf stage, we sprayed them with GR24. The expression level of *GbD14* began to increase at 5 min after spraying. Moreover, the expression was continuously upregulated for 48 h, and after 48 h, the expression peaked for a second time (Fig. 6A).

Branch development of plants requires the participation of multiple hormones. Therefore, we used a variety of hormone treatments on cotton plants to test whether the *GbD14* gene responds to those hormones. The hormones selected were auxin, cytokinin, ABA, GAs, JA, and BRs. The results showed that the expression level of *GbD14* changed in response to the hormone treatment. At 1 h after auxin treatment, the expression level of *GbD14* increased, and the expression level of *GbD14* gradually increased at 6 h after BR treatment (Fig. 6B). In all, *GbD14* could respond to SL and other plant hormones, and these hormones may participate in the branching process of cotton.

### Response of transgenic GbD14-OE Arabidopsis to SL

**Figure 7:**
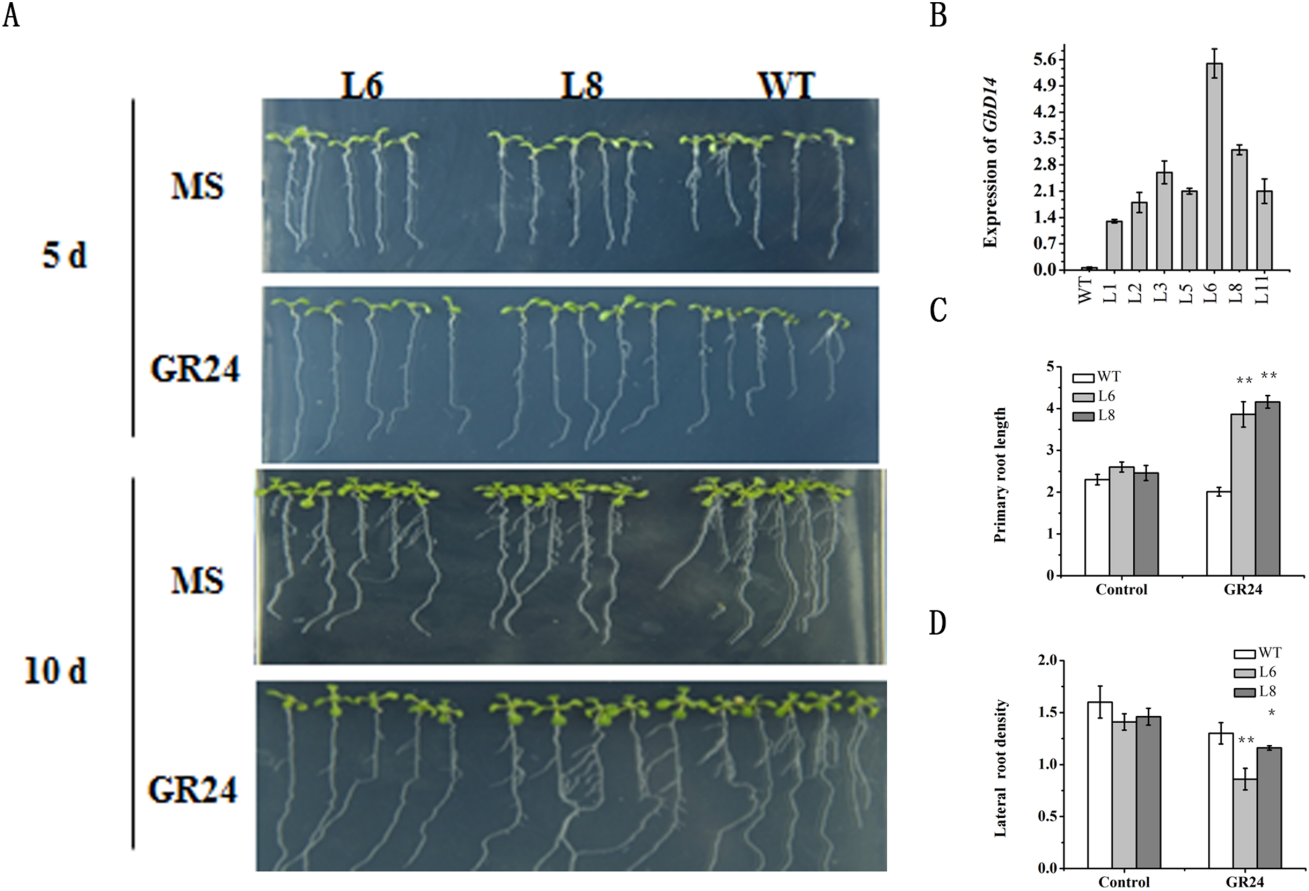
Root growth phenotype of Arabidopsis *GbD14-OE* plants. (A) Root growth of the wild type (WT), L6, and L8 on MS medium with or without GR24 . A total of 3-day-old seedlings grown on MS medium were transferred to MS medium with or without GR24. They continued to grow for 5 or 10 days before being photographed and measured. A total of 30 seedlings were measured in each experiment. (B) Expression of *GbD14* in WT and overexpression lines (L1-L11). (C and D) Comparison of primary root length (5-day seedlings) and lateral root density (10-day seedlings) among the WT, L6, and L8 on MS medium with or without GR24 (three μM). The experimental group without GR24 as Control. Experiments were performed three times with consistent results. Results are shown as mean ± SE of three replicates. The single and double asterisks show the data of the overexpression lines treat with GR24 significantly differed from that without GR24 by Student’s *t*-test at *P* ≤ 0.05 and *P* ≤ 0.01, respectively.

To further investigate the function of the *GbD14* gene, we developed transgenic *GbD14* overexpression (*GbD14-OE*) Arabidopsis plants, which we named L1-11. We then compared the expression levels of *GbD14* in these plants. qRT-PCR results showed that the expression of L6 was the highest and that the expression of L8 was moderate. These two lines were therefore selected for Arabidopsis phenotypic experiments (Fig. 7B).

To confirm the response of the GbD14 gene to SL, we first observed the root growth of Arabidopsis. A difference between the overexpression plants and the WT plants was observed. At 5 days after the GR24 treatment, the roots of L6 and L8 were longer than those of the WT (Figs. 7A and 7C). At 10 days after the GR24 treatment, the lateral root density of the overexpression and WT *A. thaliana* plants differed, and the lateral root density of L6 and L8 was significantly lower than that of the WT (Figs. 7A and 7D). In all, exogenous application of GR24 can promote the elongation of the primary roots of *A. thaliana* and decrease the density of the lateral roots. These results suggested that, compared with the WT plants, the overexpression plants could respond more quickly to SL signals.

### The cotton D14 gene rescued the Arabidopsis *d14* mutant phenotype

**Figure 8:**
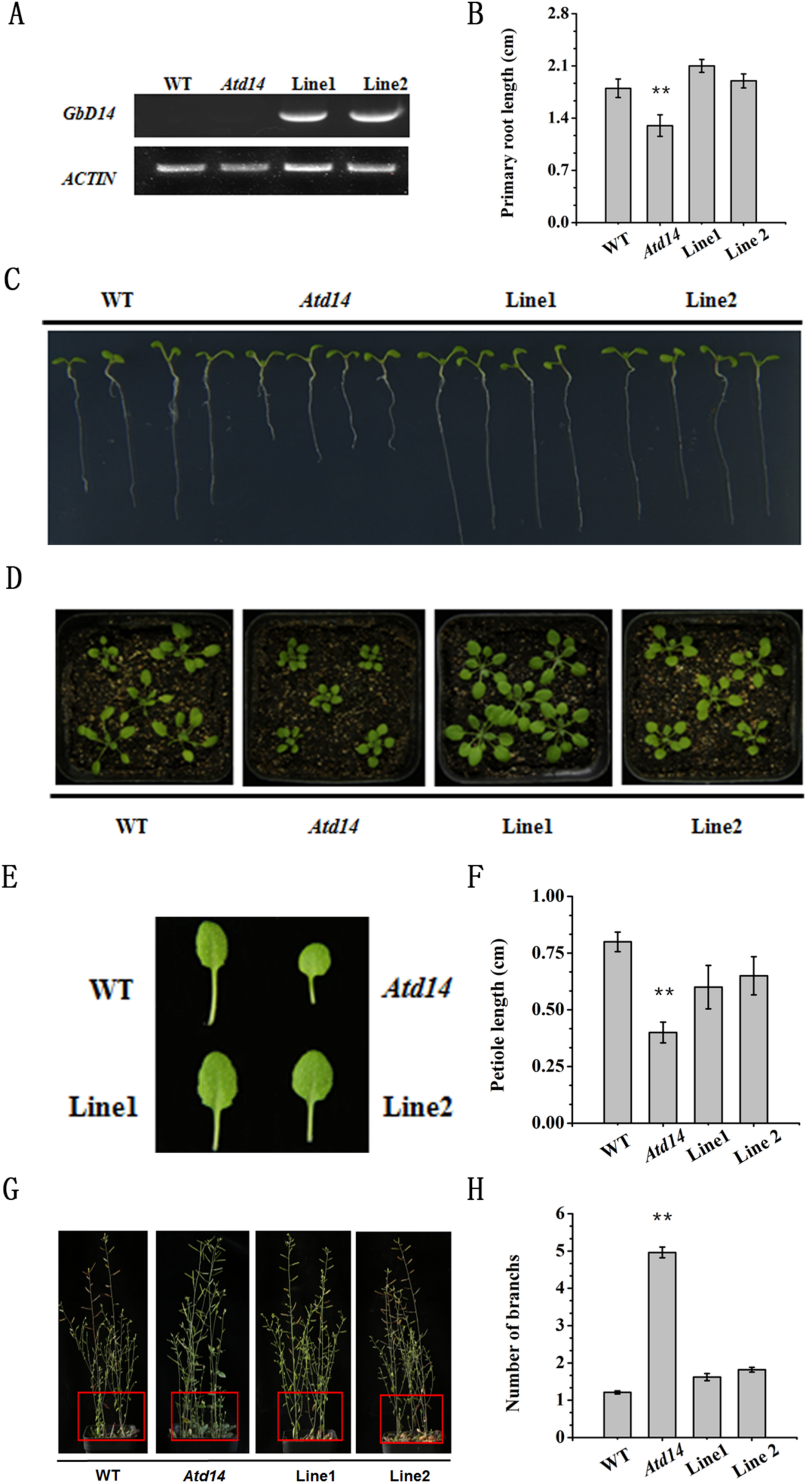
Phenotype of the recurrent line of *Atd14* mutant. (A) Analysis of the expression level of GbD14 in the complemented lines (Line1, Line2) by RT-PCR. *ACTIN* was used as the internal control. The expression level of GbD14 was detected in 7-day WT, *Atd14* mutant, Line1, Line2 seedlings. (B and C) Root growth phenotype and statistics of the strains above. A total of 3-day-old seedlings grown on MS medium were transferred to MS medium. They continued to grow for 7 days before being photographed and measured. (D) Growth phenotype of seedlings in nutrient soil. A total of 7-day-old seedlings grown on MS medium were transferred to nutrient soil. They continued to grow for 14 days before being photographed and measured. (E) Representative leaf phenotypes of seedlings in (D). (F) Quantitative analysis on the leaf length for the sixth leaves of the indicated plants. (G) Shoot branching phenotypes of Line1 and Line2 at the bolting stage. The branches of each seedling are marked with a red square. (H) Statistical analysis of branch numbers. Experiments were performed three times with consistent results. A total of 30 plants were used per replicate. Results are shown as mean ± SE of three replicates. The double asterisks show significant differences between the WT and the transgenic plants by Student’s *t*-test at *P* ≤ 0.01, respectively.

Cotton mutants are difficult to generate, and GbD14 is similar to AtD14, thus, to study the function of GbD14, we selected *Atd14*, a well-studied Arabidopsis mutant, for transformation with GbD14 and generated restored lines . After screening, two restored Arabidopsis lines, Line1 and Line2, were obtained (Fig. 8A).

The growth phenotypes of the WT, mutant and restored plants were observed. The root length of the mutant plants was shorter than that of the WT plants, however, the phenotypes of the restored plants were similar to those of the WT plants, and there were no significant difference in the lengths of the primary roots (Figs. 8B and 8C). By observing the leaf shape of the three plant types, we found that the leaves of the mutants were small and compact (Figs. 8D and 8E), and the petiole length was significantly shorter than that of WT, while the restored plants did not differ much from the WT plants (Figs. 8D–8F). When the Arabidopsis plants reached maturity, we observed an increase in the number of branches of the *Atd14* mutant. However, the number of branches between the restored plants and WT plants were similar; compared with the *Atd14* mutants, the WT plants had significantly fewer branches (Figs. 8G and 8H). In summary, the *GbD14* gene can rescue the phenotype of the *Atd14* mutant. This suggests that *GbD14* plays an important role in the development of cotton branches.

## Discussion

### GbD14 has the structure necessary for a SL receptor

In plant cells, hormone receptors are sensors that perceive external signals. D14, which acts as a receptor for SL, has an essential conserved functional domain—the α/β hydrolase domain (Bennett & Leyser, 2014). This domain has three conserved amino acid positions, Ser96, Asp217, and His246, which are prerequisites for ensuring the function of α/β hydrolases (Hamiaux et al., 2012; Sang et al., 2014). By sequence analysis and structural prediction, we found that GbD14 also contains Ser96, Asp217, and His246 amino acid sites (Fig. 1), and by homologous evolutionary analysis, we found that the GbD14 protein is closely related to the D14 proteins of other plant species, indicating that this protein is very evolutionarily conserved among different species (Fig. 1). Moreover, the results predicted by the 3D structure showed that the crystal structure of the GbD14 protein was similar to that of the DAD2 protein in *Petunia* (Hamiaux et al., 2012; Yao, Li & Xie, 2018a). It has been reported that the PhDAD2 protein is also a member of the α/β hydrolase family and acts as a receptor for SL (Yao, Li & Xie, 2018a; Yao et al., 2016; Zhao et al., 2013). All of the above results indicate that GbD14 has the structure necessary for a SL receptor. In cotton, this protein is most probably to be a receptor that senses SL signals.

### GbD14 is associated with branch development in cotton

Strigolactones are hormones that inhibit plant branching (Gomez-Roldan et al., 2008). As a receptor, D14 plays an important role in the development of plant branches. In this study, we selected two varieties, Hai 1 (normal-branch cotton) and Xinhai 16 (nulliplex-branch cotton) to detect the expression of *GbD14* in the buds, axillary buds and other tissues. It was found that GbD14 was expressed the highest in the axillary buds of normal-branch cotton varieties (Fig. 5A). It has been reported that the common form of angiosperm branch formation follows a similar process. The leaf axillary meristem gradually forms during plant growth, and after activation, it undergoes continuous cell division to form axillary buds. The growth of the axillary bud is ultimately further activated for plant branch development. As such, plants can control the formation of branches by regulating the activity of leaf axillary meristems and axillary buds (Yao, Li & Xie, 2018a). In our studies, the differential expression pattern of *GbD14* between normal-branch and nulliplex-branch cotton suggests that *GbD14* may be involved in the branch development process of cotton.

### GbD14 can sense the signal of SL and is regulated by other plant hormones

Plants branches are regulated mainly by important plant hormones such as SL. GbD14 acts as a receptor for SL and senses this signal (Yao, Li & Xie, 2018a). Therefore, we treated cotton with SL (GR24) and determined whether *GbD14* responded to SL by measuring its expression level. Further, the function of *GbD14* in cotton for the signaling of SL was analysed. Because trace amounts of hormones can quickly cause reactions in plants, nine time points were measured. The final results showed that the expression of *GbD14* was significantly upregulated within only 5 min after treatment with SL (Fig. 6A). However, although *GbD14* expression was upregulated in response to GR24 treatment, a second peak appeared at 48 h after treatment (Fig. 6A). This is a relatively long time point for the response of a receptor to hormones, and the specific mechanism for this delay is not yet clear. This phenomenon is similar to that in previous reports concerning the response mechanism of the SL receptor in Arabidopsis (Zhou et al., 2013). Those studies indicated that the signaling process of SL in plants is divided into short-distance signal transmission and long-distance signal transmission. The specific reason for this expression pattern needs to be further refined and analysed to gain a deeper understanding (Jiang et al., 2013).

Branch development in plants is a complex process that requires a combination of multiple hormones (Agharkar et al., 2007; Brewer et al., 2009; Hayward et al., 2009; Lo et al., 2008). In previous studies, auxin and cytokinin were reported to be involved, and BRs also affected the process (Cline, 1997; Shimizu-Sato & Mori, 2001). The present results showed that the expression of *GbD14* was upregulated after 1 h of auxin treatment (Fig. 6B). Auxin is involved in the branch development process in other plants. It has been reported that auxin can promote the synthesis of MAX3 and MAX4, which are involved in the SL synthesis pathway (Liang et al., 2010), so the expression of *GbD14* increases greatly when cotton is treated with auxin. Furthermore, at 6 h after treatment with BRs, the expression of *GbD14* increased gradually (Fig. 6B). As reported, a key regulatory gene in the BR signaling pathway interacts with BRC1, a downstream gene of SL, which may upregulate the expression of SL pathway-related genes (Wang et al., 2013). In summary, cotton branch development requires the participation of multiple hormones. GbD14, as a receptor for SL, interacts with the components of other hormone signaling pathways and participates in the branch development process. The underlying mechanism is still unclear, and additional research is needed.

### GbD14 can rescue the functions of the Arabidopsis mutant *Atd14*

To study the function of important genes in cotton, they are often overexpressed and transferred to the model plant Arabidopsis (Chen et al., 2017; Wang et al., 2016). In this study, two Arabidopsis GbD14 overexpression lines, L6 and L8, were screened out. Compared with WT, both overexpression lines responded to SL more quickly (Fig. 7). In addition, studies have reported that the rice D14 protein can restore the branched phenotype of the Arabidopsis *d14* mutant, suggesting functional conservation of D14 between different plant species (Yao et al., 2018b). Similarly, we transferred the cotton *GbD14* gene to the Arabidopsis *d14* mutant. The results showed that cotton *GbD14* can rescue the Arabidopsis multi-branched phenotype (Fig. 8). By observing the growth phenotypes of the WT plants, mutants and restored plants, we found that the root lengths of mutants were shorter than those of the WT plants at the seedling stage. During the growth phase, the leaves of the mutants were small and compact, and the lengths of the petioles were significantly shorter than those of the WT plants. During the maturity period, the number of branches in the mutant increased. However, compared with the WT lines, the restored lines did not significantly differ in terms of the aforementioned parameters (Fig. 8). The results of overexpression and genetic complementation experiments suggest that GbD14 is the Arabidopsis D14 functional ortholog. The above results are consistent with those of previous studies (Chevalier et al., 2014; Yao et al., 2016, 2018b). In our studies, *Atd14* (defective D14) causes more branching in Arabidopsis (Fig. 8G), while lower expression of GbD14 at axillary bud in Hai16 (with no branch) compared to Hai1 (normal-branching) (Fig. 5). That is because Hai16 has no branches and Hai1 has branches, the expression level of *GbD14* in axillary bud in Hai 16 is lower than that of in Hai 1. However, in Arabidopsis, D14 is a repressor during branch development, and thus the *Atd14* mutant has an increased phenotype of branching. The *GbD14* expression associated with cotton branching may be different from number of branch increasing in Arabidopsis, the mechanism needs further research in the future.

## Conclusions

In summary, the results of our studies showed that GbD14 may be a SL receptor that responds to SL signals in cotton. The GbD14 protein has the conserved domain necessary for a SL receptor, and the *GbD14* gene can be induced by GR24. In addition, the gene was differentially expressed in both normal-branch and nulliplex-branch cotton varieties. Compared with that in WT Arabidopsis, *GbD14* in overexpressed Arabidopsis plants responds more rapidly to GR24. Furthermore, *GbD14* can rescue the phenotype of the Arabidopsis *Atd14* (the reported SL receptor) mutant. The results showed that *GbD14* plays an important role in branch development in cotton. This report is the first to characterize the molecular mechanism of branch development involving the SL pathway and SL receptor in cotton and provides a theoretical basis for the ideal plant architecture of cotton improvement for future breeding research.

## Supplemental Information

10.7717/peerj.6901/supp-1 Supplemental Information 1 Analysis of the *GbD14* gene. (A) The nucleotide and amino acid sequences of the *GbD14* gene. (B) The amino acid composition of GbD14. Each color represents a kind of amino acid and the area represents amino acid content.

10.7717/peerj.6901/supp-2 Supplemental Information 2 Superimposion of PhDAD2 and GbD14 3D structures. Match Alignment: Score = 1,092.000. RMSD (Root-Mean-Square Deviation) = 0.647.

10.7717/peerj.6901/supp-3 Supplemental Information 3 GenBank accession numbers of D14 in different species was used for phylogenetic tree construction.

10.7717/peerj.6901/supp-4 Supplemental Information 4 Raw data for Fig. 8A (bottom).

10.7717/peerj.6901/supp-5 Supplemental Information 5 Raw data for Fig. 8A (top).

10.7717/peerj.6901/supp-6 Supplemental Information 6 Primer for Fig. 8A.

## Additional Information and Declarations

### Data Availability

The following information was supplied regarding data availability: The raw measurements are available in Fig. S1, Fig. S2 and Table S1. In addition, the GbD14 RT-PCR, ACTIN 2 RT-PCR and RT-PCR Primer are available for Fig. 8A in the Supplemental Files.
