# Supplementary figures and images for: Functional analysis of the GbDWARF14 gene associated with branching development in cotton
Source: PeerJ. 2019 May 14;7:e6901. doi: 10.7717/peerj.6901 (PMC6524629; doi:10.7717/peerj.6901)

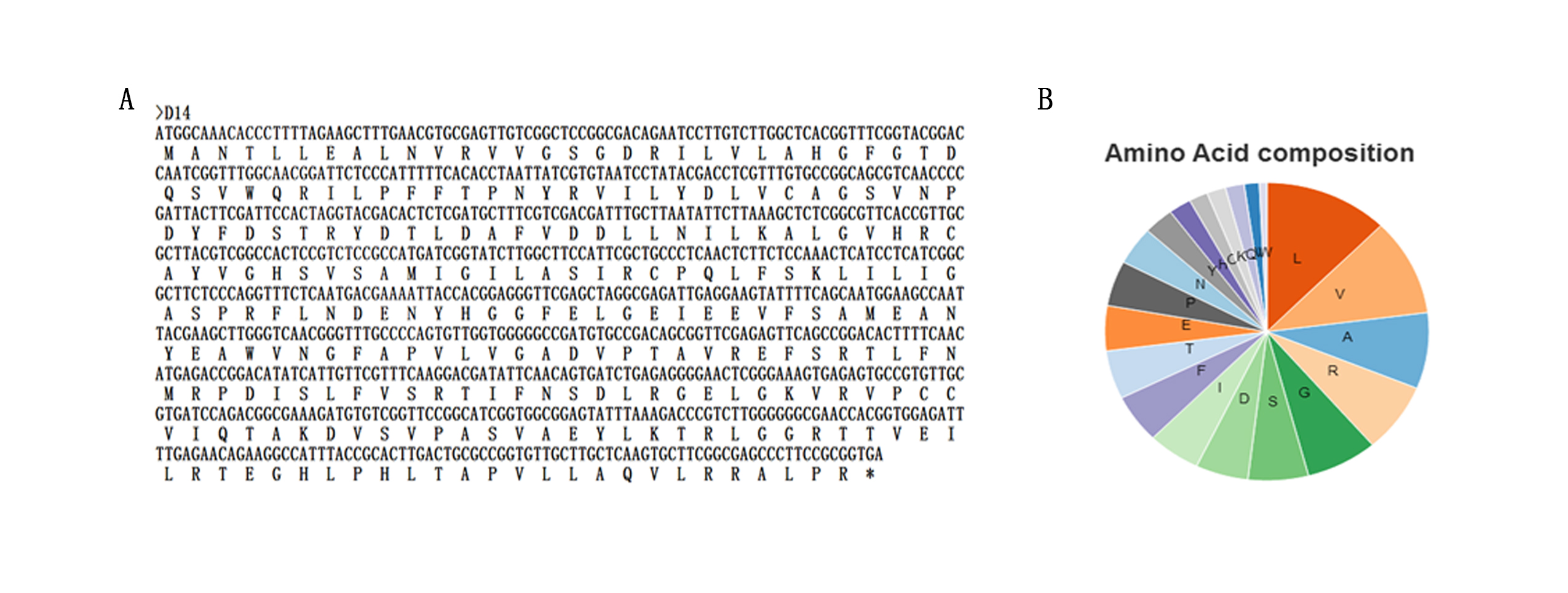

Supplement: Supplemental Information 1 — (A) The nucleotide and amino acid sequences of the GbD14 gene. (B) The amino acid composition of GbD14. Each color represents a kind of amino acid and the area represents amino acid content. [file peerj-07-6901-s001.jpg]

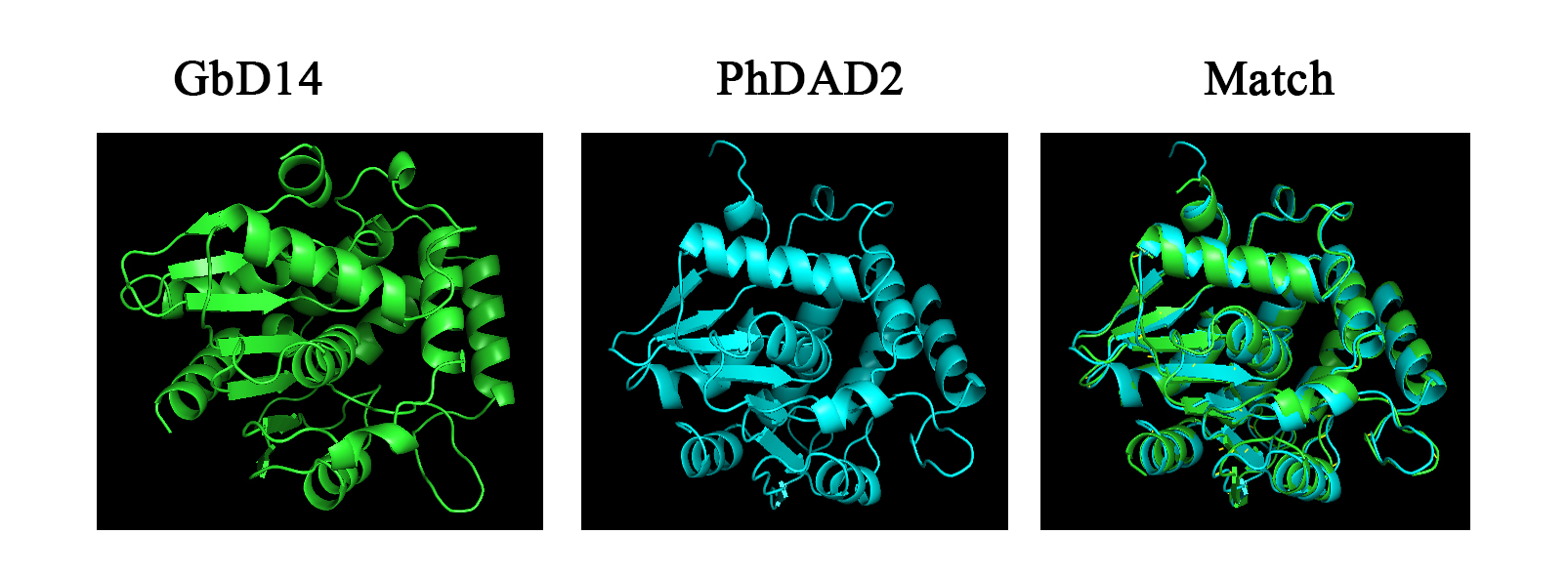

Supplement: Supplemental Information 2 — Match Alignment: Score = 1,092.000. RMSD (Root-Mean-Square Deviation) = 0.647. [file peerj-07-6901-s002.jpg]

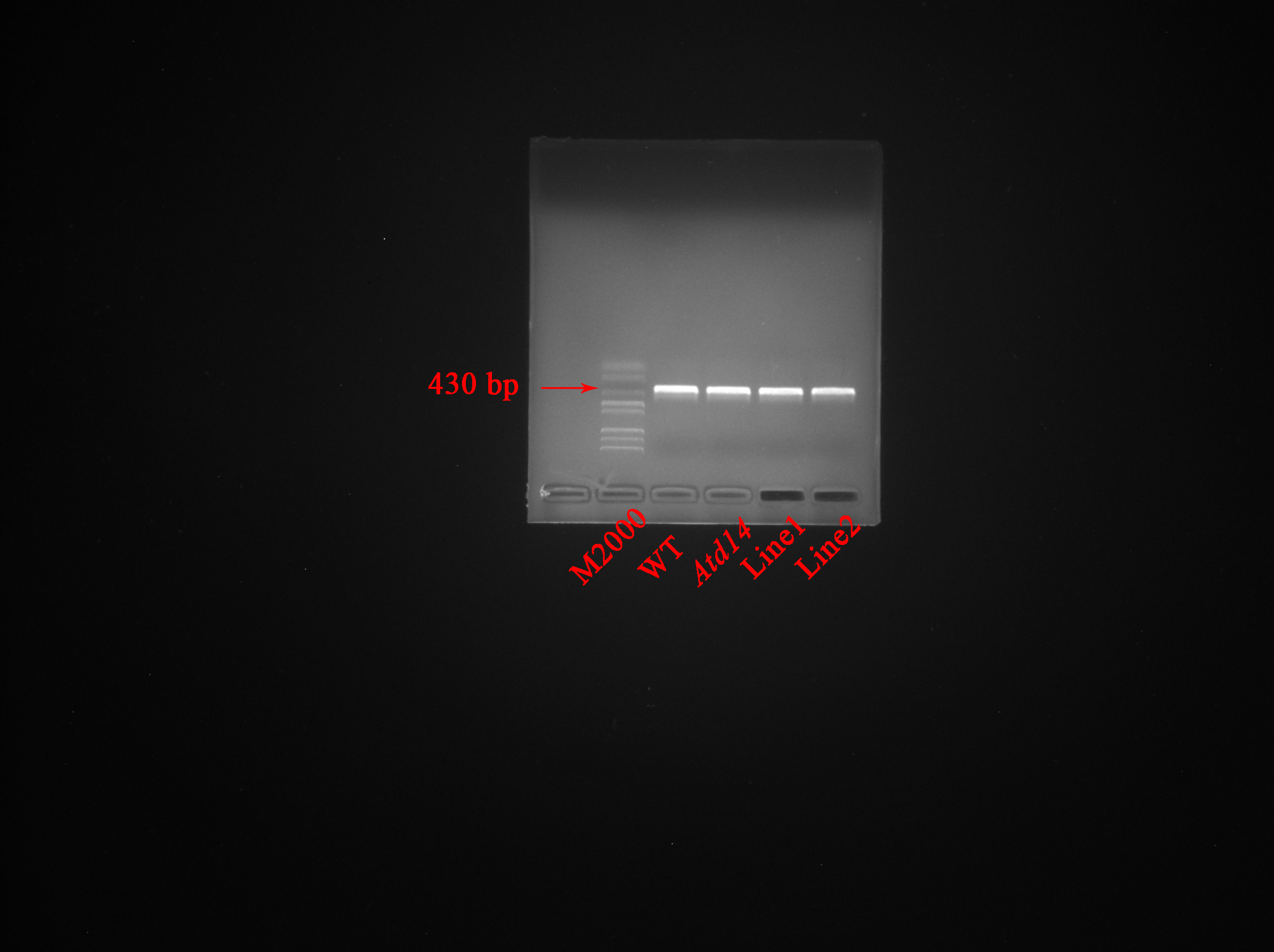

Supplement: Supplemental Information 4 [file peerj-07-6901-s004.jpg]

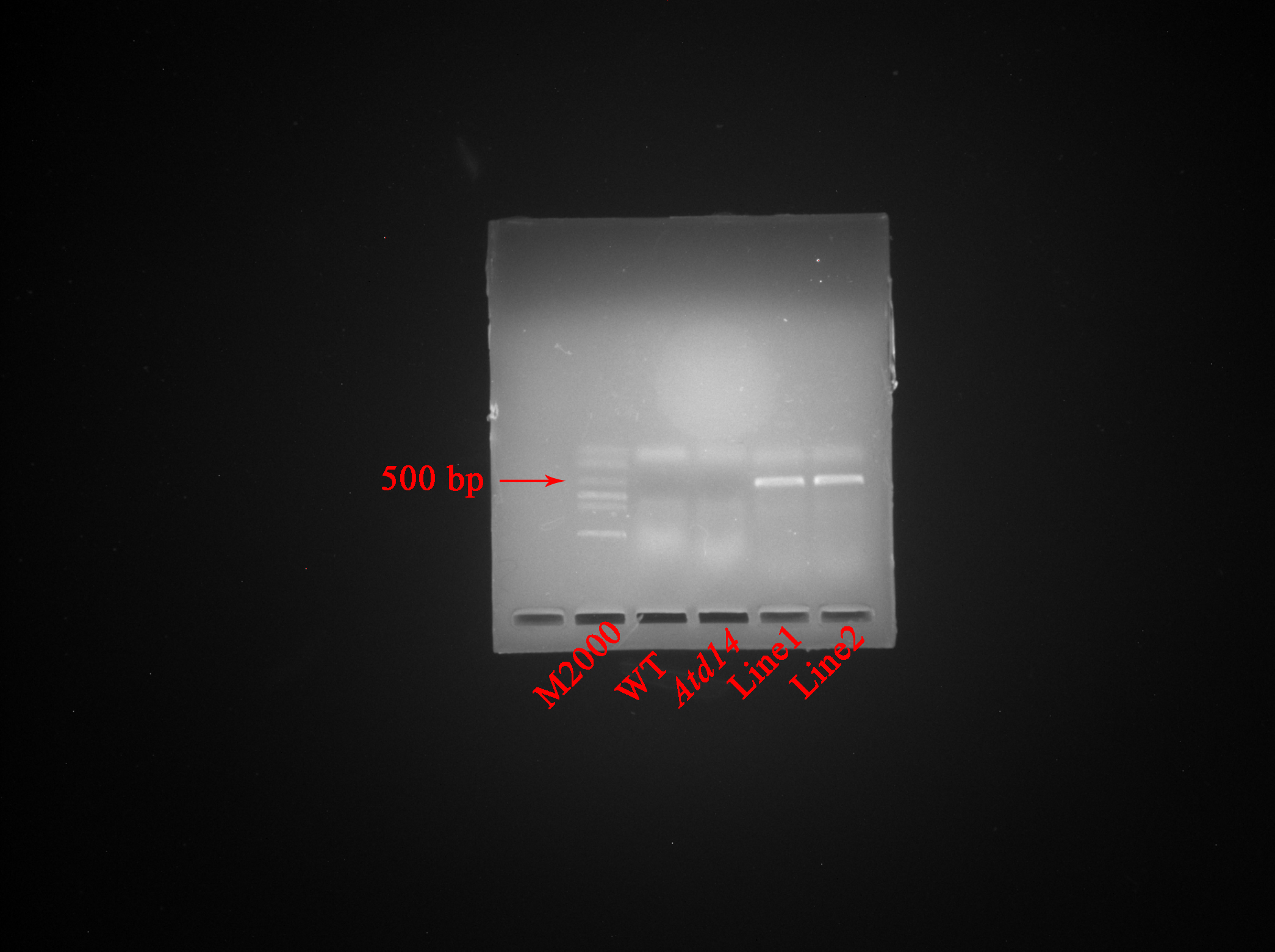

Supplement: Supplemental Information 5 [file peerj-07-6901-s005.jpg]
