# Supplementary material for: Functional analysis of the GbDWARF14 gene associated with branching development in cotton
Source: PeerJ. 2019 May 14;7:e6901. doi: 10.7717/peerj.6901 (PMC6524629; doi:10.7717/peerj.6901)
Supplement: Supplemental Information 3 [file peerj-07-6901-s003.docx]

Supplement Table 1

Table 1 Accession numbers of D14 in difference species was used for phylogenetic tree construction

| Name | Species | GenBank ID |
| --- | --- | --- |
| ZmD14 | *Zea mays* | XP_008660429.1 |
| AtD14 | *Arabidopsis thaliana* | NP_195463.1 |
| BnD14 | *Brassica napus* | XP_013731709.1 |
| CpD14 | *Carica papaya* | XP_021893497.1 |
| CsD14 | *Camelina sativa* | XP_010517275.1 |
| DzDAD2 | *Durio zibethinus* | XP_022752585.1 |
| GbD14 | *Gossypium barbadense* | AKH05365.1 |
| GhD14 | *Gossypium hirsutum* | XP_016717728.1 |
| GmD14 | *Glycine max* | NP_001347076.1 |
| GrD14 | *Gossypium raimondii* | XP_012451974.1 |
| HbDAD2 | *Hevea brasiliensis* | XP_021676516.1 |
| HvD14 | *Hordeum vulgare subsp. vulgare* | AJP07999.1 |
| NaDAD2 | *Nicotiana attenuata* | XP_019258478.1 |
| OsD14 | *Oryza sativa Japonica Group* | XP_015631400.1 |
| PhDAD2 | *Petunia x hybrida* | AFR68698.1 |
| PsDAD2 | *Populus euphratica* | XP_011017119.1 |
| PtDAD2 | *Populus trichocarpa* | XP_002302409.1 |
| RcDAD2 | *Ricinus communis* | XP_002510101.1 |
| RsD14 | *Raphanus sativus* | XP_018438965.1 |
| TcD14 | *Theobroma cacao* | XP_007018509.2 |
